# The potential of large language model chatbots for application to epilepsy: Let’s talk about physical exercise^☆^

**DOI:** 10.1016/j.ebr.2024.100692

**Published:** 2024-06-29

**Authors:** Rizia Rocha-Silva, Bráulio Evangelista de Lima, Geovana José, Douglas Farias Cordeiro, Ricardo Borges Viana, Marília Santos Andrade, Rodrigo Luiz Vancini, Thomas Rosemann, Katja Weiss, Beat Knechtle, Ricardo Mario Arida, Claudio Andre Barbosa de Lira

**Affiliations:** a Faculty of Physical Education and Dance, Federal University of Goiás, Goiânia, Brazil; b Faculty of Information and Communication, Federal University of Goiás, Goiânia, Brazil; c Institute of Physical Education and Sports, Federal University of Ceará, Fortaleza, Brazil; d Department of Physiology, Federal University of São Paulo, São Paulo, Brazil; e Center for Physical Education and Sports, Federal University of Espírito Santo, Vitória, Brazil; f Institute of Primary Care, University of Zurich, Zurich, Switzerland; g Medbase St. Gallen Am Vadianplatz, St. Gallen, Switzerland

**Keywords:** Physical exercise, Artificial intelligence, Chatbot

## Abstract

In this paper, we discuss how artificial intelligence chatbots based on large-scale language models (LLMs) can be used to disseminate information about the benefits of physical exercise for individuals with epilepsy. LLMs have demonstrated the ability to generate increasingly detailed text and allow structured dialogs. These can be useful tools, providing guidance and advice to people with epilepsy on different forms of treatment as well as physical exercise. We also examine the limitations of LLMs, which include the need for human supervision and the risk of providing imprecise and unreliable information regarding specific or controversial aspects of the topic. Despite these challenges, LLM chatbots have demonstrated the potential to support the management of epilepsy and break down barriers to information access, particularly information on physical exercise.

## Highlights

• LLMs have the potential to revolutionize access and communication in healthcare.
• AI chatbots can aid in managing epilepsy through guidance on physical exercise.
• ChatGPT provides conservative information for contact sports in epilepsy.
• LLMs are promise, but their limitations should not be ignored.

## Introduction

The last two years have been marked by significant advances in artificial intelligence (AI), particularly in its natural language processing (NLP) applications such as large-scale language models (LLMs) [1]. This evolution has allowed solutions and algorithms previously restricted to programmers and developers to become accessible to anyone [2]. The combination of easy-to-use interfaces and large collections of subject-specific information has contributed to the popularity of generative AI through chatbots. A chatbot is a computer program designed to simulate conversation with human users, usually over the internet. The primary goal of a chatbot is to engage in conversation with users, recognize their queries and requests, and provide relevant and helpful responses [3]. ChatGPT by Open AI, Gemini by Google, Copilot Chat by Microsoft, and Luzia on WhatsApp and Telegram are some of the more popular chatbots currently available. The majority of these programs are powered by AI and NLP technologies.

The applicability of these tools has been explored in the healthcare field, where a range of studies have evaluated the reliability of information provided by chatbots [3], [4]. Research spanning a variety of medical specialties has investigated their use. This includes their adoption by medical students [5], the provision of information on ambulatory practices [6], and addressing myths and misconceptions about cancer [7], cancer in specific regions such as the neck [8], and spinal surgery [6]. However, to date, there has been little research on the use of AI chatbots for the dissemination of epilepsy information [9], [10], [11], [12].

## How can AI be used to facilitate the management of epilepsy?

Epilepsy is a complex neurological condition, the diagnosis and treatment of which may be positively impacted by the use of AI [13]. In this context, LLMs have the potential to revolutionize access and communication in healthcare by offering significant benefits to the daily self-management of people with epilepsy (PWEs). Through user friendly interfaces and easy handling, chatbots based on LLMs consolidate information on a single platform, making health knowledge more personalized. While traditional NLP focuses on identifying, selecting, and grouping clinical information, including seizure frequency, documented conditions in PWEs, and EEG classifications [14], [15]. LLMs have a greater capacity for text generation that allows them to engage in complex in-depth dialogs, from which they continuously learn [16].

Using a simple smartphone command, individuals can ask questions, receive detailed answers, and explore different aspects through LLMs, enhancing understanding and awareness of certain condition [12]. This incorporation of technology empowers PWEs to adopt healthier lifestyle choices, such as improved nutrition and regular physical exercise [17], [18], [19], [20].

It is noteworthy that AI-based chatbots are a possible tool for overcoming barriers to communication and access to information. Unlike healthcare professionals or specialized websites, many of which are readable only in English (which can be an important barrier for non-English speakers), AI chatbots are able to collate information from multiple sources and deliver that information in different languages [2]. This functionality provides access to a broader range of knowledge and facilitates more intuitive interactions with advanced technology [21]. In low- and middle-income countries, where public health faces challenges due to limited resources, including issues with the availability of accessible and inaccurate information, chatbots emerge as a crucial tool [22]. These regions house 80 % of PWEs, 75 % of whom lack adequate treatment [23]. A recent study by Boßelmann, Leu, and Lal [12] investigated the use of LLMs through the ChatGPT 3.5 in the management of epilepsy. In tests simulating patients, ChatGPT 3.5 provided correct information about medication side effects and action plans for acute seizures, aligned with medical guidelines and recommendations. However, in more specific or controversial epilepsy topics, ChatGPT made mistakes (incorrect suggestions for epilepsy surgery or relationship between variants in SCN9A and autosomal dominant epilepsy).

## The use of chatbots to manage epilepsy through physical exercise

For many years, there were misconceptions regarding the practice of physical exercise by PWEs [24], [25]. However, more recent scientific literature has exhaustively demonstrated the benefits of physical exercise for PWEs [17], [18], [26]. These benefits span a range of parameters, including physical and mental health, social integration, reduction of stress markers and epileptiform EEG activity, and the number of seizures [17], [18]. Nevertheless, the proportion of PWEs who are insufficiently active remains high [18], [19]. Indeed, misconceptions and myths about the effects of physical exercise on this population contribute to the perpetuation of this situation. For example, a widely held misconception is the belief that physical exercise is harmful for PWEs, under the argument that it could increase the likelihood of seizures. Furthermore, there is a widespread belief that engaging in sports can elevate the risk of falls, resulting in a greater susceptibility to injuries. Another persistent myth is the supposed incompatibility between PWEs and participation in contact sports, aquatic activities, or performing intense physical exercise [27].

**Fig. 1:**
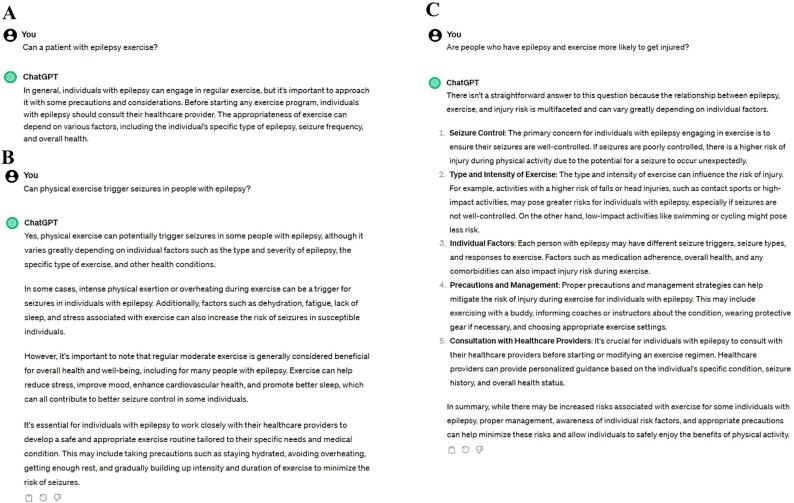
Output with ChatGPT 3.5 answers about the myths surrounding physical exercise for people with epilepsy.

We asked ChatGPT 3.5 about the myths regarding the relationship between epilepsy and physical exercise. The first of the three questions was related to the inclusion of PWEs in physical exercise, and the consequences that were attributed to the practice (Fig. 1).

We questioned whether PWEs can do physical exercise. ChatGPT replied that participating in physical exercise it is possible, but it requires the supervision of a health professional (Fig. 1A). Regarding the myth about physical exercise triggering seizures, ChatGPT stated that physical exercise can potentially trigger seizures, especially with intense physical exertion, but emphasized that moderate exercise can be beneficial for PWEs (Fig. 1B). While the model's conclusion emphasizes the benefits of physical exercise for PWEs, its initial response might lead the reader to believe that physical exercise triggers seizures, and that its high intensity can be a contributing factor, which is not supported by current scientific literature [18], [26].

We subsequently inquired about injuries through physical activity. ChatGPT correctly responded about the relationship of injury not being directly related to physical exercise, but rather to a set of factors, which ChatGPT exemplified in its response (Fig. 1C). Studies suggest that PWEs have a higher risk of injuries, which can occur whether they engage in physical exercise or sport [27].

**Fig. 2:**
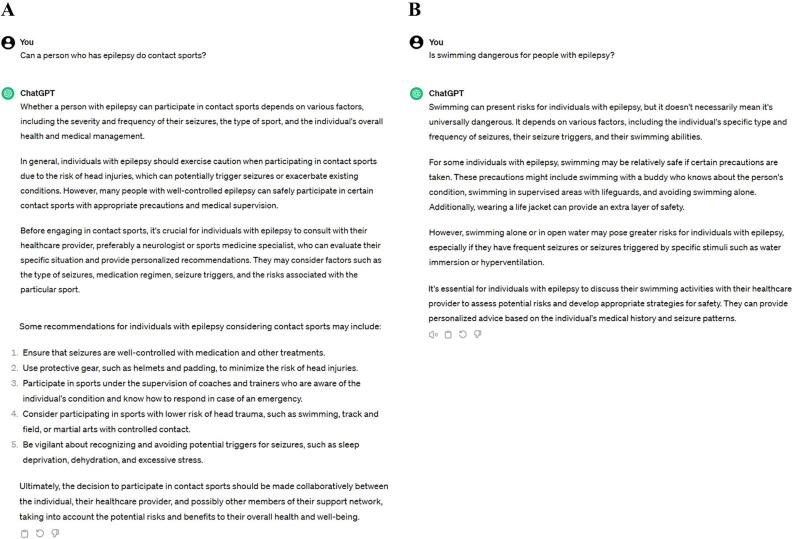
ChatGPT 3.5 responses on the engagement in contact sports (A) and swimming (B) for people with epilepsy.

Subsequently, inquiries were made regarding sports historically deemed hazardous or restricted for PWEs (Fig. 2).

Despite its conservative approach, ChatGPT 3.5 provided accurate information about the possibility of practicing sporting activities, such as swimming and contact sports, detailing the necessary care that aligns with those described by the ILAE Task Force on Sport and Epilepsy. Briefly, the ILAE categorizes sports according to the risk associated with the practice: Group 1 (no significant additional risk for PWEs); Group 2 (moderate risks for PWEs); and Group 3 (high risks for PWEs), considering the probability of a seizure occurring, its type and the usual moment of the seizure, among other factors [26].

The ILAE Task Force on contact sports delineates two classifications: Group 1, encompassing most collective contact sports (for example, judo and wrestling); and Group 2 which includes contact sports that involve potentially serious injury (for example, boxing, karate, and Muay Thai) (Fig. 2A). For swimming and aquatic activities that do not necessitate deep diving, the modality is classified under Group 2 (moderate risks for PWEs) (Fig. 2B) [26].

Although LLMs promise to improve the health of PWEs, they face challenges, such as limited knowledge about recent events and specialized topics, risks of social bias inherent in the training data, which can lead to inconsistencies and false predictions (hallucinations). Moreover, because they are trained on text data from the internet, LLMs can also produce outputs that reflect societal biases [28]. Finally, it is important to consider the potential limitation of LLMs for working with structured data, such as data available in spreadsheets. These systems may also have performance difficulties in specific languages, which can exacerbate accuracy issues. Despite these hurdles, the continuous learning capability of LLMs, supported by ongoing updates and refinements, promises gradual improvements. This could lead to delivering more accurate and specialized information [22]. In this context, methodologies have been proposed to enhance AI learning, ensuring data accuracy and security. These include supervised learning interventions that provide corrective feedback to AI systems during their training [29] and alignment-tuning, which typically involves instructing learning through preference tuning via reinforcement learning from human feedback. This enables large language models (LLMs) to act in accordance with human intentions and values, potentially establishing expert feedback loops [29], [30]. Possible solutions to those issues involve fine-tuning to reduce the tendency for hallucinations, inserting relevant knowledge in the prompt, providing access to external search engines, filtering biased content from the training data, and including more content from other languages [31].

We can no longer ask if AI will enhance healthcare and access to information, but rather when it will do so [31]. Given this, we believe that chatbots integrated with AI can be useful for PWEs who are interested in starting physical exercise program, previously accompanied by health professionals. Currently, we do not recommend using the platform for suggesting or designing physical exercise programs, due to limitations in its capabilities in specific area [32]. Future studies are necessary to elucidate the quality of a wider range of issues regarding the relationship between epilepsy and physical exercise. This includes evaluating other LLM programs. Additionally, it is essential to explore possibilities related to digital literacy, aiming to increase engagement and improve people accessibility to new technologies related to AI.

## Conclusion

Recent evidence from a range of fields has demonstrated the reliability of AI-based chatbots. This reliability is enhanced by their internet integration and access, and their ability to learn from each interaction. These tools show potential for applications with specific diseases including epilepsy and may help to alleviate concerns about the safety of physical exercise and provide educative material on its benefits to those with epilepsy. Although LLMs show promise, their limitations should not be ignored, as current programs can provide misleading or inaccurate responses to queries. However, the adaptability of LLMs and their constant updates are expected to increase their accuracy over time. It is also important to state that these AI technologies are not intended to replace patient consultations with healthcare professionals, and necessary caution and human supervision in the use of the information provided should be ensured due to the limitations and possible biases of these technologies.

## Ethical statement

The manuscript did not require ethical committee approval as it did not involve human subjects and/or animals.

## CRediT authorship contribution statement

**Rizia Rocha-Silva:** Writing – review & editing, Writing – original draft, Conceptualization. **Bráulio Evangelista de Lima:** Writing – review & editing, Writing – original draft, Formal analysis, Conceptualization. **Geovana José:** Writing – review & editing, Writing – original draft. **Douglas Farias Cordeiro:** Writing – review & editing, Writing – original draft. **Ricardo Borges Viana:** Writing – review & editing, Writing – original draft, Visualization. **Marília Santos Andrade:** Writing – review & editing, Writing – original draft, Visualization. **Rodrigo Luiz Vancini:** Writing – review & editing, Writing – original draft. **Thomas Rosemann:** Writing – review & editing, Writing – original draft. **Katja Weiss:** Writing – review & editing, Writing – original draft. **Beat Knechtle:** Writing – review & editing, Writing – original draft. **Ricardo Mario Arida:** Writing – review & editing, Writing – original draft, Conceptualization. **Claudio Andre Barbosa de Lira:** Writing – review & editing, Writing – original draft, Validation, Supervision, Funding acquisition, Formal analysis, Data curation, Conceptualization.

## Declaration of competing interest

The authors declare that they have no known competing financial interests or personal relationships that could have appeared to influence the work reported in this paper.

## References

[1] Liu Y., Han T., Ma S., Zhang J., Yang Y., Tian J. (2023). Summary of ChatGPT-Related research and perspective towards the future of large language models. Meta-Radiol.

[2] Teubner T., Flath C.M., Weinhardt C., van der Aalst W., Hinz O. (2023). Welcome to the Era of ChatGPT et al.: the prospects of large language models. Bus Inf Syst Eng.

[3] Chakraborty C., Pal S., Bhattacharya M., Dash S., Lee S.S. (2023). Overview of Chatbots with special emphasis on artificial intelligence-enabled ChatGPT in medical science. Front Artif Intell.

[4] Ayers J.W., Poliak A., Dredze M., Leas E.C., Zhu Z., Kelley J.B. (2023). Comparing physician and artificial intelligence chatbot responses to patient questions posted to a public social media forum. JAMA Intern Med.

[5] Cascella M., Montomoli J., Bellini V., Bignami E. (2023). Evaluating the feasibility of ChatGPT in healthcare: an analysis of multiple clinical and research scenarios. J Med Syst.

[6] Liu J., Wang C., Liu S. (2023). Utility of ChatGPT in clinical practice. J Med Internet Res.

[7] Johnson S.B., King A.J., Warner E.L., Aneja S., Kann B.H., Bylund C.L. (2023). Using ChatGPT to evaluate cancer myths and misconceptions: artificial intelligence and cancer information. JNCI Cancer Spectr.

[8] Wei K., Fritz C., Rajasekaran K. (2024). Answering head and neck cancer questions: An assessment of ChatGPT responses. Am J Otolaryngol.

[9] Daungsupawong H., Wiwanitkit V. (2024). ChatGPT’s responses to questions related to epilepsy. Seizure Eur J Epilepsy.

[10] Tirumala A.K.G., Mishra S., Trivedi N., Shivakumar D., Singh A., Shariff S. (2024). A cross-sectional study to assess response generated by ChatGPT and ChatSonic to patient queries about Epilepsy. Telemat Informatics Reports.

[11] Kim H.-W., Shin D.-H., Kim J., Lee G.-H., Cho J.W. (2024). Assessing the performance of ChatGPT’s responses to questions related to epilepsy: A cross-sectional study on natural language processing and medical information retrieval. Seizure - Eur J Epilepsy.

[12] Boßelmann C.M., Leu C., Lal D. (2023). Are AI language models such as ChatGPT ready to improve the care of individuals with epilepsy?. Epilepsia.

[13] An S., Kang C., Lee H.W. (2020). Artificial Intelligence and Computational Approaches for Epilepsy. J Epilepsy Res.

[14] Yew A.N.J., Schraagen M., Otte W.M., van Diessen E. (2023). Transforming epilepsy research: A systematic review on natural language processing applications. Epilepsia.

[15] Xie K., Gallagher R.S., Conrad E.C., Garrick C.O., Baldassano S.N., Bernabei J.M. (2022). Extracting seizure frequency from epilepsy clinic notes: a machine reading approach to natural language processing. J Am Med Inform Assoc.

[16] Peng C., Yang X., Chen A., Smith K.E., PourNejatian N., Costa A.B. (2023.1–10.). A study of generative large language model for medical research and healthcare. Npj Digit Med.

[17] Johnson E.C., Helen Cross J., Reilly C. (2020). Physical activity in people with epilepsy: A systematic review. Epilepsia.

[18] Arida R.M. (2021). Physical exercise and seizure activity. Biochim Biophys Acta - Mol Basis Dis.

[19] Van Den Bongard F., Hamer H.M., Sassen R., Reinsberger C. (2020). Sport and physical activity in epilepsy: a systematic review. Dtsch Arztebl Int.

[20] Arida R.M., Scorza C.A., Schmidt B., De Albuquerque M., Cavalheiro E.A., Scorza F.A. (2008). Physical activity in sudden unexpected death in epilepsy: much more than a simple sport. Neurosci Bull.

[21] Nazir A., Wang Z. (2023). A Comprehensive survey of ChatGPT: advancements, applications, prospects, and challenges. Meta-Radiology.

[22] Wang X., Sanders H.M., Liu Y., Seang K., Tran B.X., Atanasov A.G. (2023). ChatGPT: promise and challenges for deployment in low- and middle-income countries. Lancet Reg Heal West Pacific.

[23] Epilepsy. World Health Organization 2024. https://www.who.int/news-room/fact-sheets/detail/epilepsy (accessed April 3, 2024).

[24] Vancini R.L., de Lira C.A.B., Gomes da Silva S., Scorza F.A., da Silva A.C., Vieira D. (2010). Evaluation of physical educators’ knowledge about epilepsy. Arq Neuropsiquiatr.

[25] Vancini R.L., Benedito-Silva A.A., Sousa B.S., Da Silva S.G., Souza-Vancini M.I., Vancini-Campanharo C.R. (2012). Knowledge about epilepsy among health professionals: a cross-sectional survey in Sao Paulo, Brazil. BMJ Open.

[26] Capovilla G., Kaufman K.R., Perucca E., Moshé S.L., Arida R.M. (2016). Epilepsy, seizures, physical exercise, and sports: A report from the ILAE Task Force on Sports and Epilepsy. Epilepsia.

[27] Pimentel J., Tojal R., Morgado J. (2015). Epilepsy and physical exercise. Seizure.

[28] van Diessen E., van Amerongen R.A., Zijlmans M., Otte W.M. (2024). Potential merits and flaws of large language models in epilepsy care: a critical review. Epilepsia.

[29] Aldoseri A., Al-Khalifa K.N., Hamouda A.M. (2023). Re-thinking data strategy and integration for artificial intelligence: concepts, opportunities, and challenges. Appl Sci.

[30] Naveed H, Khan AU, Qiu S, Saqib M, Anwar S, Usman M, et al. A Comprehensive Overview of Large Language Models 2023.

[31] Landais R., Sultan M., Thomas R.H. (2024). The promise of AI Large Language Models for Epilepsy care. Epilepsy Behav.

[32] Dergaa I., Ben Saad H., El Omri A., Glenn J., Clark C., Washif J. (2024). Using artificial intelligence for exercise prescription in personalised health promotion: A critical evaluation of OpenAI’s GPT-4 model. Biol Sport.

